# Small-Molecule:Polymer Composites for Transparent Films with Visible Emission

**DOI:** 10.1007/s10895-024-03880-w

**Published:** 2024-08-13

**Authors:** Enrique Pérez-Gutiérrez, Franciso D. Calvo, Miriam F. Beristain, Perumal Venkatesan, Subbiah Thamotharan, M. Judith Percino

**Affiliations:** 1 Unidad de Polímeros y Electrónica Orgánica, Instituto de Ciencias, Benemérita Universidad Autónoma de Puebla, Val3-Ecocampus Valsequillo, Independencia O2 Sur 50, San Pedro Zacachimalpa, Puebla Mexico; 2 Decanatura de Ingenierías, Universidad Popular Autónoma de Puebla, 21 Sur #1103, Barrio de Santiago, Puebla, C.P 72410 Mexico; 3 Department of Chemistry, Srimad Andavan Arts and Science College (Autonomous), Tiruchirappalli, 620 005 India; 4 Biomolecular Crystallography Laboratory, Department of Bioinformatics, School of Chemical and Biotechnology, SASTRA Deemed University, Thanjavur, 613 401 India

**Keywords:** Polyvinylcarbazole, Acrylonitrile derivatives, Red shift emission, Exciplex, OLEDs

## Abstract

The analysis of the shift in photoluminescence emission for a blend of polyvinylcarbazole and acrylonitrile derivative compounds is reported. The small-molecule compounds have different functional groups, phenyl, pyridine, or methyl phenyl, attached to an acrylonitrile group. According to the functional group, the blue emission for pure dye shifts to green or yellowish in the blend film. Several PVK:dye ratios from 0:100 to 20:80 were used for film deposition. The film morphology was analyzed by atomic force microscopy; for low dye content, homogeneous films were achieved. However, aggregates of several micrometers are formed on the surface of films with higher dye concentrations. The shift in emission occurs only with PVK, and for a non-conjugated matrix such as polystyrene, the emission remains unchanged. The interaction of dyes with PVK leading to change in emission was also achieved by grinding dye and polymer. Results showed that shifts in emission could come from exciplex formation along with changes in dye intermolecular interactions. The blend films were highly transparent in the visible spectra due to the absorption in the UV region for dye and matrix. The films with ratio PVK: dye ratio 80:20 was used as active layer in OLEDs.

## Introduction

Organic fluorescent materials remain an important research issue owing to their wide variety of applications in sensors, bioimaging, and optoelectronic devices [1–7]. The study and comprehension of recombination processes such as fluorescence, phosphorescence, exciplex, and, recently, thermally activated delayed fluorescence have allowed for improving the emission properties of organic compounds, especially the fluorescence quantum yield [8–12]. In addition, it is well known that the emission wavelength (*λ*_*em*_) for organic materials can be tailored by means of functional groups, with electron donor or acceptor behavior, attached to a π-conjugated unit [13, 14]. Moreover, the emission properties of organic compounds depend not only on chemical structure but also on intermolecular interactions. Such interactions arise from molecular aggregation and can cause either concentration quenching or aggregation-induced emission [15–17]. It is known that the molecular packing motifs such as herringbone as well as slipped or lamellar π-stacking influence these intermolecular interactions [18]. In turn, noncovalent interactions like hydrogen bonds O−H⋅⋅⋅O, N−H⋅⋅⋅O and O−H⋅⋅⋅N which appear in molecules with polar substituents play a fundamental role in crystal engineering. At the same time weak interactions such as like C−H⋅⋅⋅O, C−H⋅⋅⋅N, C−H⋅⋅⋅π are driving forces that can play a significant role in the crystal packing in the absence of strong hydrogen bond interactions. Both strong and weak noncovalent interactions can modify the optical properties and specially the fluorescence emission in organic conjugated compounds [19–23]. When organic fluorescent molecules are embedded in a polymer matrix to form film, its intermolecular interactions and optical properties are also modified. For instance, F. Ito et al., reported the change in fluorescence spectra of a pyrene derivative when embedded in a poly(vinylalcohol) matrix [24]. They reported a redshift in emission with increasing dye concentration into the polymer matrix and was assigned to a dimer or excimer-like emission, i.e. to the formation of aggregates in the films. Also, the emission of difluoroboron avobenzone complex could be tunned as function of its concentration in a poly(methylmethacrylate) matrix [25]. We also have reported the tunning in emission, from blue to orange, for a acrylonitrile derivative embedded in a polyvinylcarbazole (PVK) matrix. For the use of polymer matrix, the change in emission can also arise from exciplex formation. The exciplex is a complex excited stated that occurs in a system formed by molecules with donor and acceptor behavior; in this complex, one of the molecules is in the excited state, and the other is in the ground state [26, 27]. 

We recently conducted a study on the correlation between the molecular structure-optical properties of π-conjugated acrylonitrile derivatives, and the important role of molecular packing and intermolecular interactions in forming polymorphs with different emission properties [28, 29]. Our studies focused on stimulus affecting the emission wavelength of acrylonitrile derivatives; such factors can be external stimuli, [30–32] internal structure properties, [22, 23, 33–35] as well as the interactions with polymer matrix to form exciplex emission [36, 37]. We were able to tune the emission for small-molecule acrylonitrile derivatives from blue to orange by embedding them in a polyvinyl carbazole (PVK) matrix [36]. To continue our study about the effect of polymer matrix on small-molecule emission properties, we have prepared thin films based on acrylonitrile derivatives showing intense blue emission, which have been incorporated into the PVK matrix. The blended films showed a redshift in emission compared with the dye; the shift was observed even for films where dye segregation was evidenced by atomic force microscopy. Electrochemical measurements were conducted to calculate the HOMO level for the dyes and then calculate the exciplex energy. The deposited films were highly transparent in the visible range because both polymer and dye have absorption below 400 nm, while the emission was above 500 nm. The films with more significant redshifts in emission and better morphology were used as active layers in OLEDs.

## Experimental

**Scheme 1:**
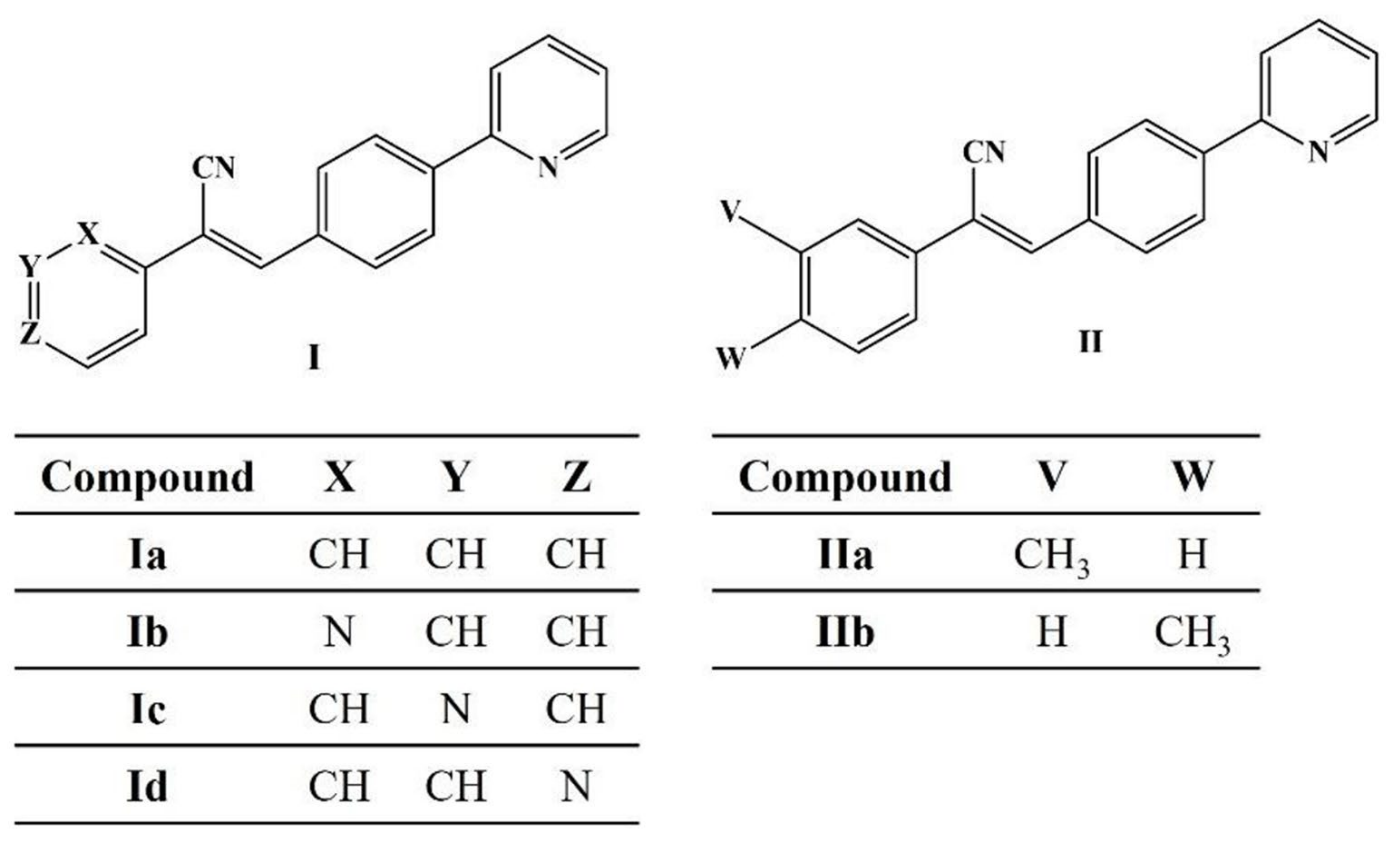
Structure of acrylonitrile derivatives

The synthesis and physiochemical characterization of acrylonitrile derivatives has been previously reported [28, 29, 38]. Briefly, compound **Ia** was synthesized by the condensation of 4-(2-pyridyl)benzaldehyde with phenylacetonitrile, while isomers **Ib**-**Id** were obtained from condensation of 4-(2-pyridyl)benzaldehyde with 2, 3, or 4- pyridylacetonitrile; compounds **IIa**-**IIb** were synthesized from the condensation of 4-(2-pyridyl)benzaldehyde with 3-or 4-methylbenzylcyanide (Scheme 1). In all cases KOH was used as catalyst. All compounds were purified by recrystallization from methanol or ethyl acetate.

### Thin Films and OLEDs

Polyvinylcarbazole and polystyrene (PS) from Aldrich were used as the polymer matrix; the polymer and acrylonitrile derivative compounds were dissolved in chlorobenzene. PVK:dye ratios of 0:100, 20:80, 40:60, 50:50, 60:40, and 80:20% w/w were used; meanwhile, for PS:dye film, the ratio was 80:20. For all the solutions, the concentration was 10 mg/ml. Thin films were deposited by the spin coating technique onto glass substrates for optical and morphological analysis. OLEDs were prepared with glass/ITO as substrate and anode, and a 40 nm film of Poly(3,4-ethylenedioxythiophene)-poly(styrenesulfonate) (PEDOT:PSS) from Heraeus was used as the hole transport layer. The active layer was deposited by the spin coating technique from a solution PVK:dye with a ratio of 80:20; the active layer was 120 nm. This ratio was chosen according to morphological characterization, which shows the formation of a homogeneous film with no dye segregation. As a cathode, 150 nm of silver was thermally evaporated. Cyclic voltammetry (CV) of the samples was recorded with a potentiostat Autolab PGSTAT128N, from Metrohm. As working electrode and counter electrode a Pt wire was used and Ag/AgCl was the reference electrode, measure was conducted at a constant scan rate of 100 mV/s. All potentials in this work are presented in comparison to the Fc+/Fc (ferrocene) reference potential.

#### Characterization

The absorption spectra were acquired with a spectrometer SD2000 (Ocean Optics) and a UV/Vis DT 1000 CE light source (Analytical Instrument Systems, Inc. NJ). The absorption in solution for pure compounds was recorded with chlorobenzene as the solvent in solutions with a concentration of 10^− 5^ M. For photoluminescence (PL) and electroluminescence (EL) were measured with an Ocean Optics USB4000 spectrophotometer. For PL UV lamp (UVGL-58) with emission at a 365 nm was used as the excitation source. The fluorescence quantum yield was measured on the FLS1000 of Edinburgh Instruments equipment, which is equipped with a 450 W Xe lamp and an integrating sphere. The same equipment was used to acquire the average lifetime profiles; in this case, a 375 nm pulsed laser was used as the excitation source. The morphological analysis was conducted with an atomic force microscope (AFM) NanoSurf EasyScan.

## Results

For compound **Ia**, we have previously reported changes in fluorescence emission achieved by solvent treatment; compound **Ia** crystallized in a triclinic or orthorhombic crystal system according to the solvent used [28]. Solvent treatment leads to changes in color, crystal habit, and fluorescent emission properties. The emission for the as-synthesized compound has a maximum emission wavelength (*λ*_*em*_) of 454 nm; meanwhile, for the compound recrystallized with ethyl acetate, the *λ*_*em*_ was at 492 nm. The changes in emission and quantum yield efficiency were assigned to the features of the molecular packing in the single crystal that led to enhanced or decreased π-π interactions owing to a change in face-to-face alignment in the molecules [28]. However, for compounds **Ib**-**Id** and **IIa**-**IIb**, achieving the same behavior with solvent treatment was not possible, even when several solvents were tried. Gierschner and Park have highlighted the implications of intermolecular arrangements such as H- or J-aggregation, herringbone or π-stacking, the occurrence of excimers, size effects, and polycrystallinity on the solid-state optoelectronic properties of distyrylbenzenes [39]. On the other hand, when organic fluorescent compounds are embedded in a polymer matrix, their optoelectronic properties can also be modified owing to exciplex formation and small-molecule interactions with polymer chains.

**Fig. 1:**
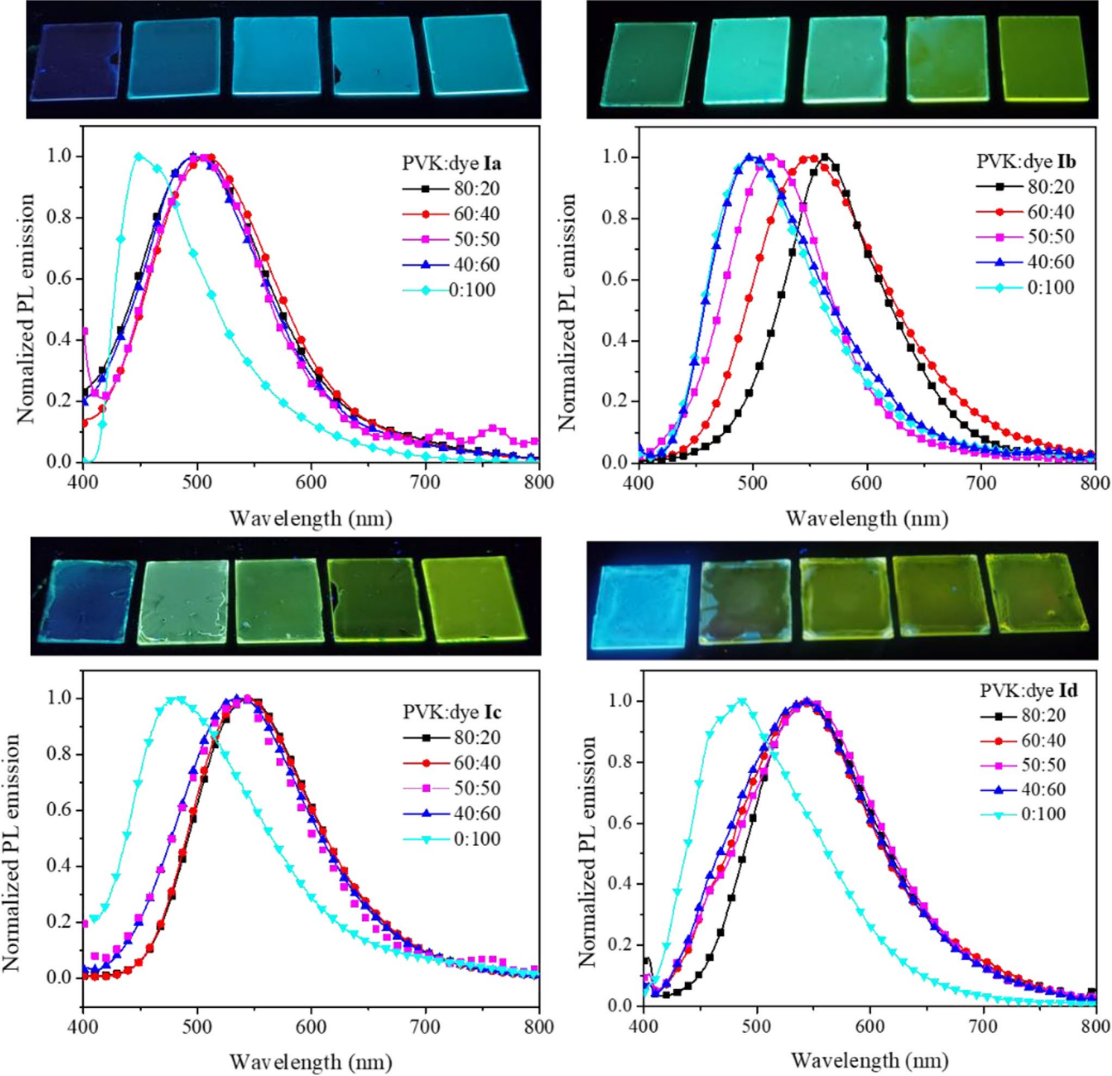
Emission spectra for film PVK:dye **I**, at different components ratio

To evaluate optoelectronic properties of acrylonitrile derivatives Films were prepared for compounds **Ia**-**Id** and **IIa**-**IIb**, by using PVK as matrix. The emission for films based on the blend PVK-dye showed a redshift compared to pure components. For compounds **Ia**-**Id**, the shift in wavelength of maximum emission (*λ*_*em*_) differed for the kind of substituent attached to the acrylonitrile group (Fig. 1). A significant shift was observed for the phenyl substituent (compound **Ia**) where the pure compound deep-blue emission at 450 nm shifted to 510 nm (blue-green) when embedded in PVK at 20% w/w. For pyridine substituent, the shift was quite similar for the three isomers from 480 to 498 nm up to 564 nm, Table 1.

Along with the shift in emission wavelength, the photoluminescence quantum yield (PLQY) slightly increased when compounds were embedded into the PVK matrix. The PLQY for compounds **Ia**-**Id** was 6.45, 14.21, 14.96, and 7.49, respectively, and increased to 11.34, 20.74, 21.04, and 11.81 for the PVK: dye ratio of 80:20% w/w.

**Fig. 2:**
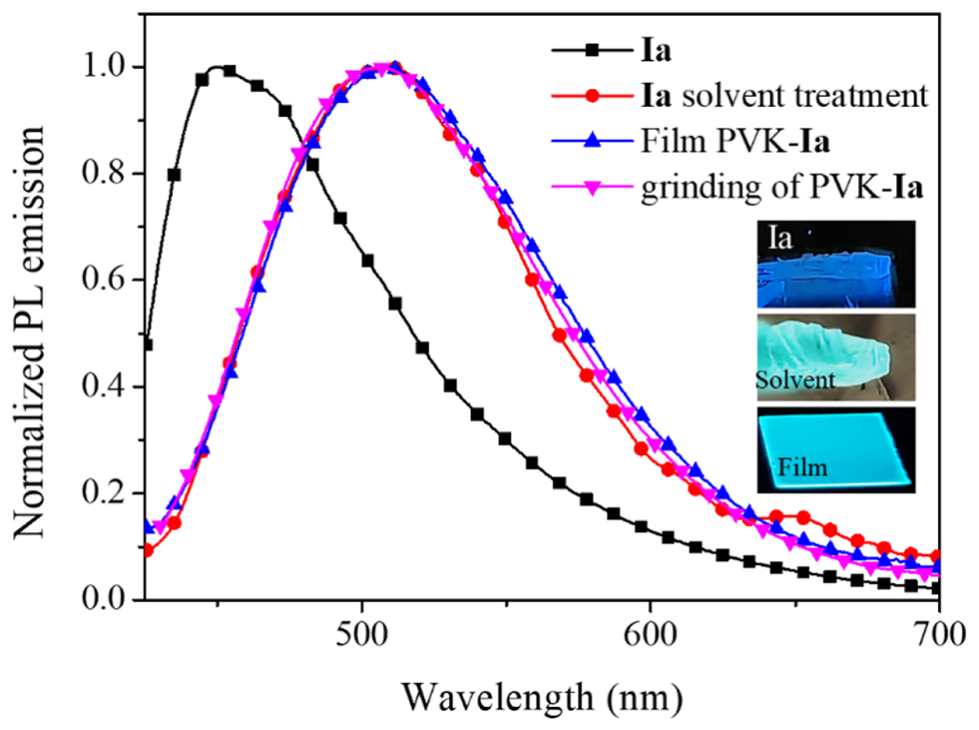
Photoluminescence spectra for compound **Ia**, the compound after solvent treatment, the emission in a film with PVK as the matrix, and the PVK-dye grinding

Interestingly, the shift in emission for compound **Ia** reached by embedding it in the polymer matrix, is like the reported with solvent treatment (Fig. 2) [28]. This indicates that intermolecular interactions of **Ia** owing to solvent treatment, could be also induced by the PVK matrix. Despite for compounds **Ic**-**Id** there was not possible to achieve changes in emission properties with solvent treatment, the shift was observed when embedded in the polymer matrix.

**Fig. 3:**
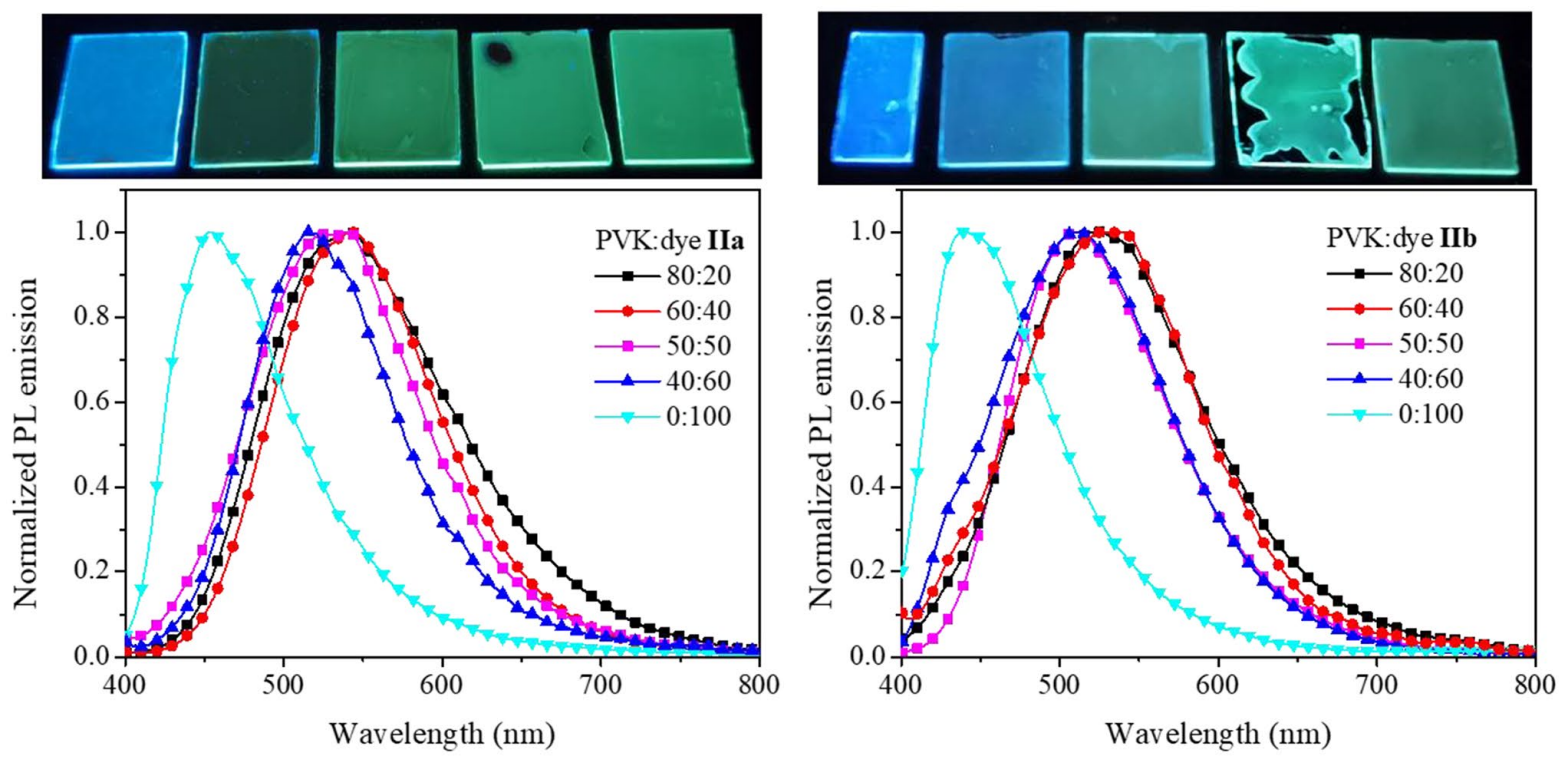
Emission spectra for film PVK: dye **II**, at different components ratio

For compounds **IIa** and **IIb**, with the methylphenyl group as substituent, the shift in *λ*_*em*_ was from 454 nm up to 519–540 nm and from 439 nm to 512–530 nm (Fig. 3), respectively. In this case, the shift was slightly higher, about 86–91 nm, compared with compounds **Ia**-**Id**, with a 60–66 nm displacement. However, because the emission of pristine dyes **IIa**-**IIb** is blue, its emission within PVK film is at a longer wavelength than the observed for **Ia**-**Id** dyes. We have previously studied the intermolecular and non-covalent interactions presented in the crystal structures of isomers **I** and **II** and the influence of such interactions on optical properties and HOMO and LUMO levels [29, 38]. 

**Fig. 4:**
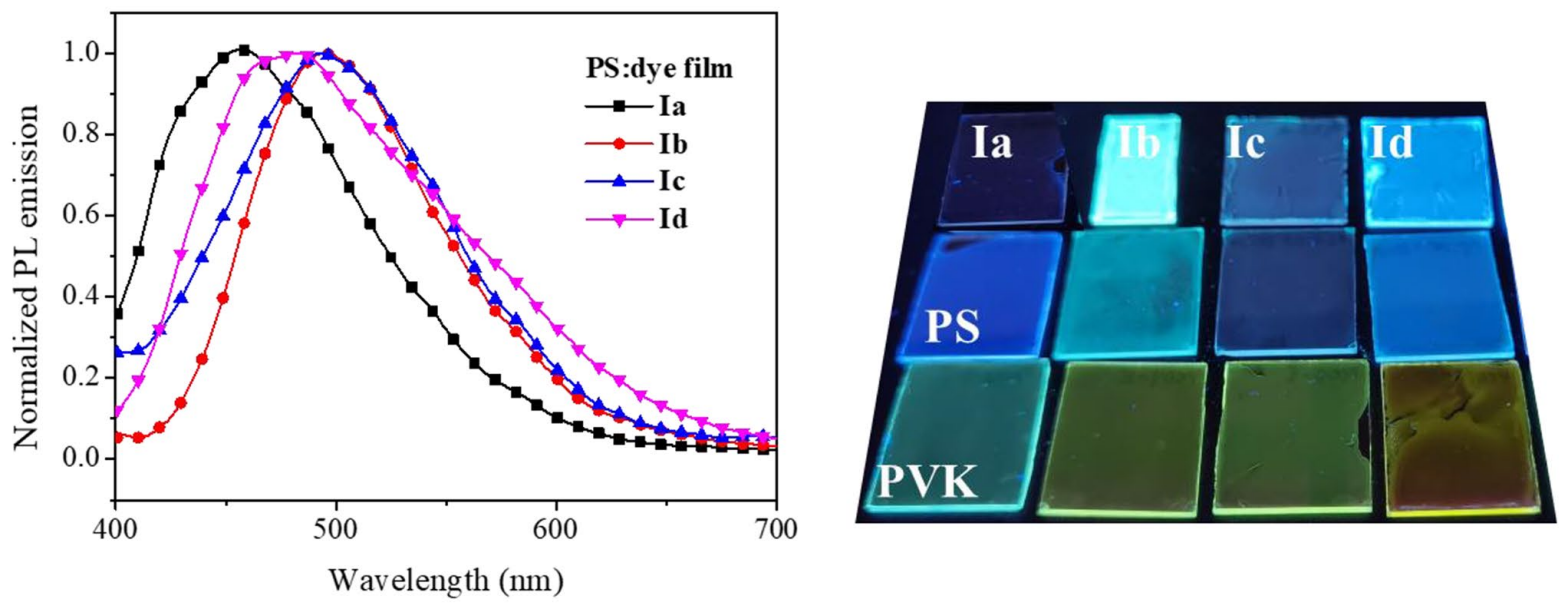
Emission spectra for films with dyes **Ia**-**Id** and polystyrene as matrix. Image of pure dyes and embedded in PS or PVK

However, the shift in emission is not observed when a non-conjugated matrix is used. Figure 4 shows the emission spectra and picture for polystyrene (PS) based films with an 80:20 polymer:dye ratio. For PS: dye films, the emission is similar to pure dye films at 456 nm, 496 nm, 493 nm, and 481 nm for the dyes **Ia**-**Id**, respectively. The picture shows that the shift in emission is achieved only when PVK is used as the matrix.

According to our previous studies about intermolecular interactions owing to solvent treatment that affect the emission of compound **Ia** and the observed changes in emission for films with PVK and PS as the matrix; the changes in emission could be due to modification in the π-π interactions for the dyes. The shift in emission, owing to interactions with the PVK matrix, was also achieved by grinding dye and polymer. For the obtained powder the emission spectra were like the recorded for films, as shown in Fig. 2 for compound **Ia**.

The use of PVK could also induce the exciplex formation between polymer and dyes. It has been reported that the energy of the exciplex can be approached from the difference in energy of the highest occupied molecular orbital (HOMO) of hole transport material (PVK) and the lowest unoccupied molecular orbitals of the electron transport material (dye). [40]^,^ [41].

**Fig. 5:**
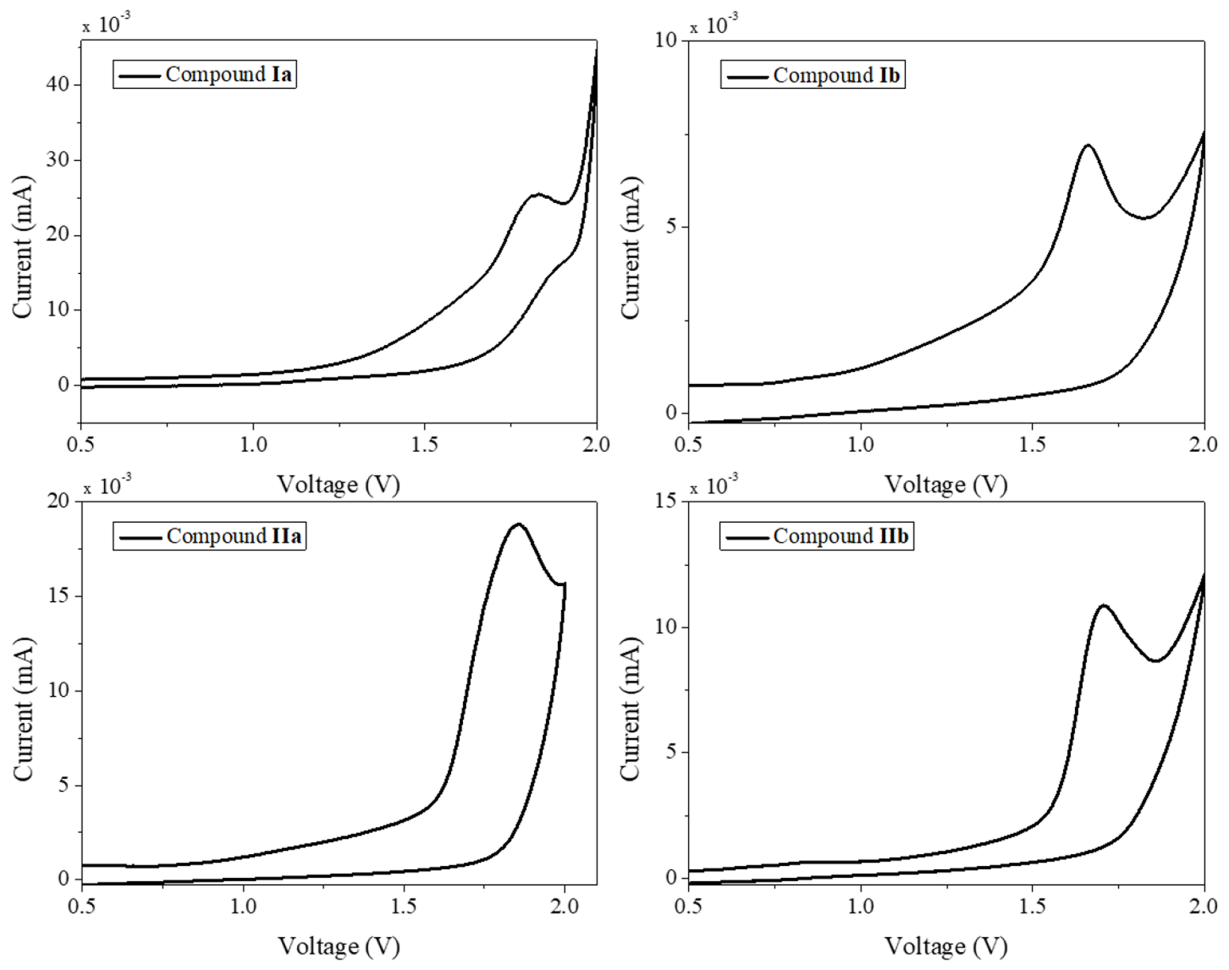
Voltammograms of compounds **Ia**, **Ib**, **IIa**, and **IIb**

Voltammograms for compounds **Ia** and **Ib** are shown in Fig. 5 (voltammograms for compounds **Ic** and **Id** were very similar to the recorded for **Ib**). The used value for Fc+/Fc was − 5,1 eV, [42] hence, the value for HOMO of compound **Ia** is -6.64 eV, meanwhile for compounds **Ib**, **Ic**, and **Id**, were about − 6.76 eV; these values are summarized in Table 1. For compounds **IIa** and **IIb**, the HOMO energy level was − 6.68 eV and − 6.63 eV, respectively.

The LUMO value for the dyes was calculated from the following equation:

1 $${E_{LUMO}} = {\rm{ }}{E_{HOMO}} + {\rm{ }}{E_g}^{Optic}$$

**Fig. 6:**
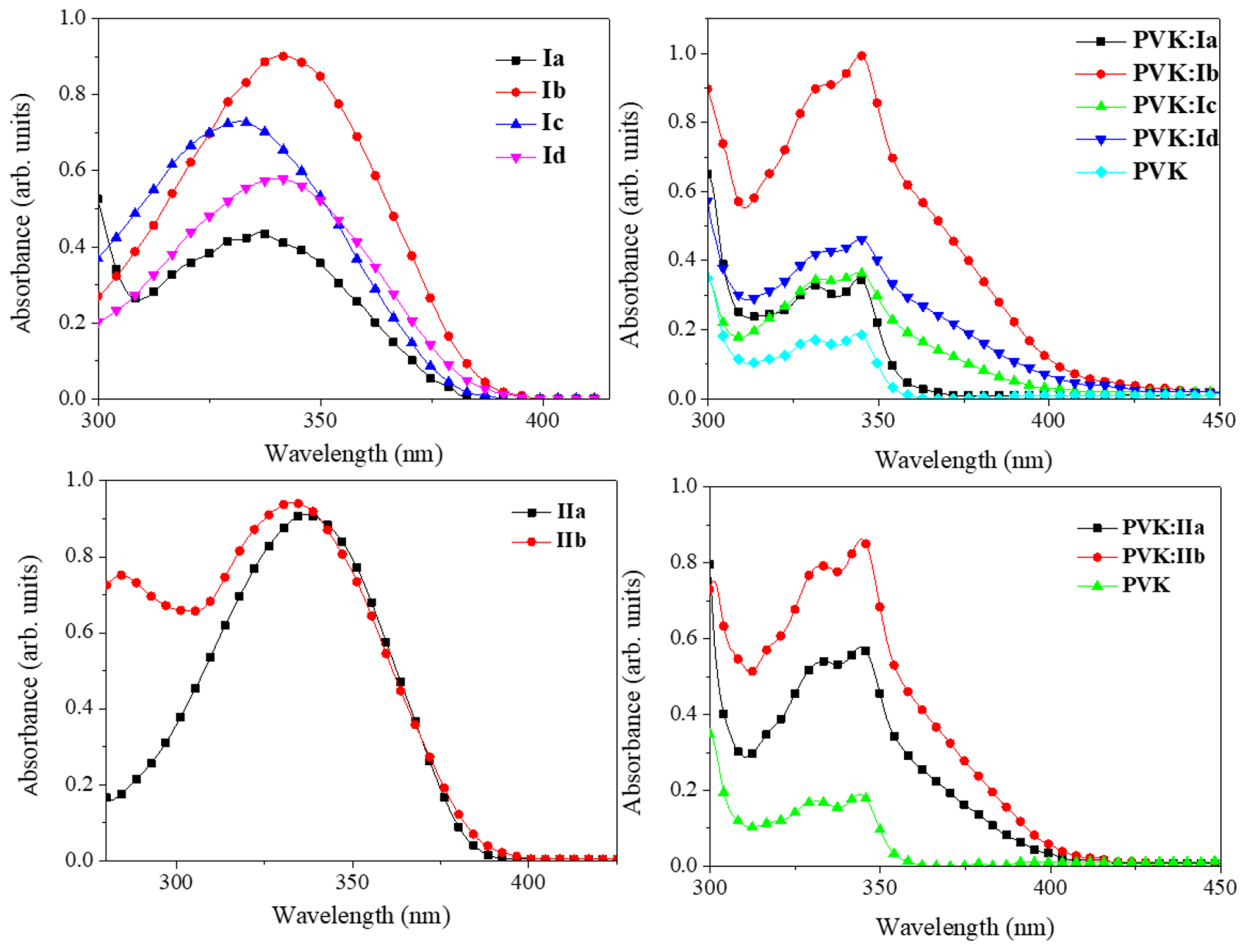
Absorption spectra for the dyes in solution (left) and for films PVK:dye (right), at ratio 80:20% w/w

Where E_g_^Optic^ is the optical band gap taken at the onset of the corresponding UV-Vis absorption spectrum (Fig. 6), the values for LUMO of dyes are about − 3.3 to -3.5 eV and are summarized in Table 1.

The HOMO of PVK is reported at about − 5.4, -5.64 eV, [43, 44] with these values and the calculated for the LUMO of dyes, the energy of exciplex emission was 2.49 eV for compound **Ia** and 2.29 for compound **Ib**-**Id**. These values are well approached to the wavelength of maximum emission for the films.

**Table 1:** Optical parameters for the films PVK: dye

	λ_em_ (nm) PVK:dye					Optical bandgap	HOMO (eV)	LUMO (eV)	Exciplex energy (eV)
	0:100	40:60	50:50	60:40	80:20				
**Ia**	450	498	505	510	510	3.27	-6.64	-3.37	2.49
**Ib**	498	501	515	549	564	3.21	-6.76	-3.55	2.29
**Ic**	480	536	543	546	546	3.27	-6.74	-3.47	2.37
**Id**	484	545	545	545	545	3.23	-6.72	-3.49	2.35
**IIa**	454	516	529	540	540	3.24	-6.68	-3.44	2.40
**IIb**	439	510	510	530	530	3.23	-6.63	-3.40	2.44

**Fig. 7:**
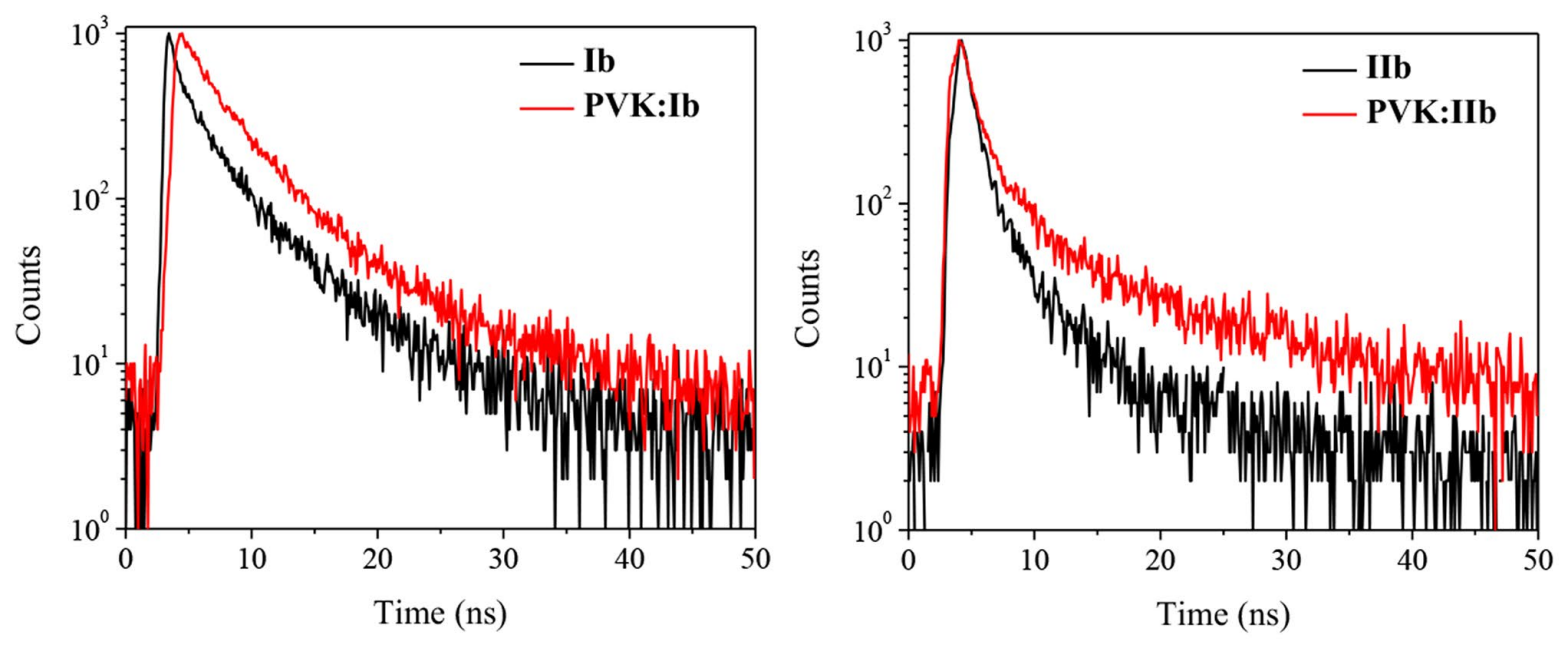
Photoluminescence decay curves for films of **Ib** and **IIb** and for the blend with PVK

Time-resolved photoluminescence decay measurements were conducted for compounds **Ib** and **IIb**, which showed the larger redshift (Fig. 7). The fluorescence lifetime (*τ*_em_) for blend film slightly increased compared with the pure compound films. The average lifetime for compounds **Ib** and **IIb** are 5.1 and 5.3 ns, meanwhile for the blend films are 7.67 and 5.98 ns. These phenomena have been reported for exciplex formation [40]. 

**Fig. 8:**
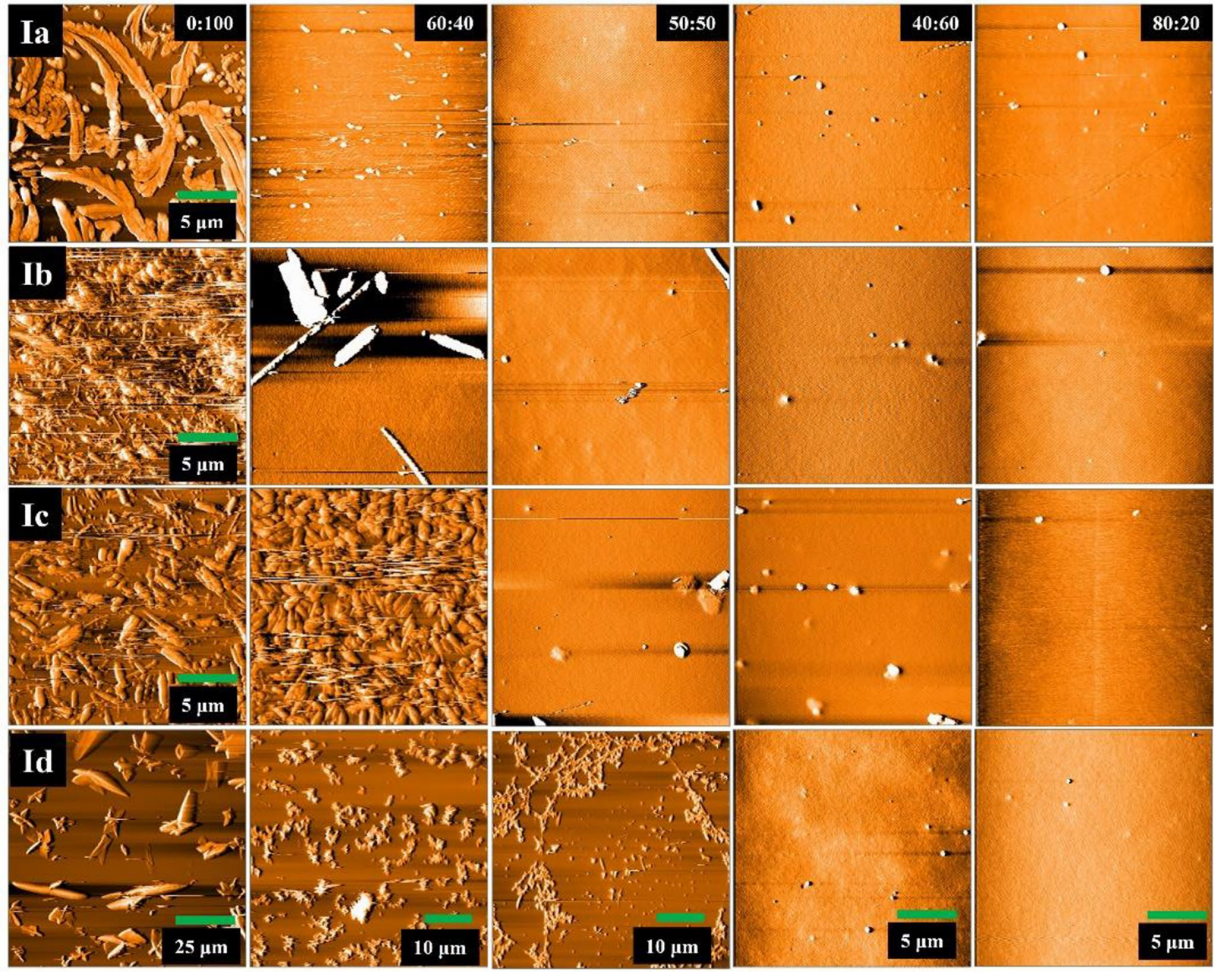
AFM images for film morphology of PVK:dye **I** film, the scanned area is 20 × 20 μm, except for the films with a higher concentration of **Id** compound

**Fig. 9:**
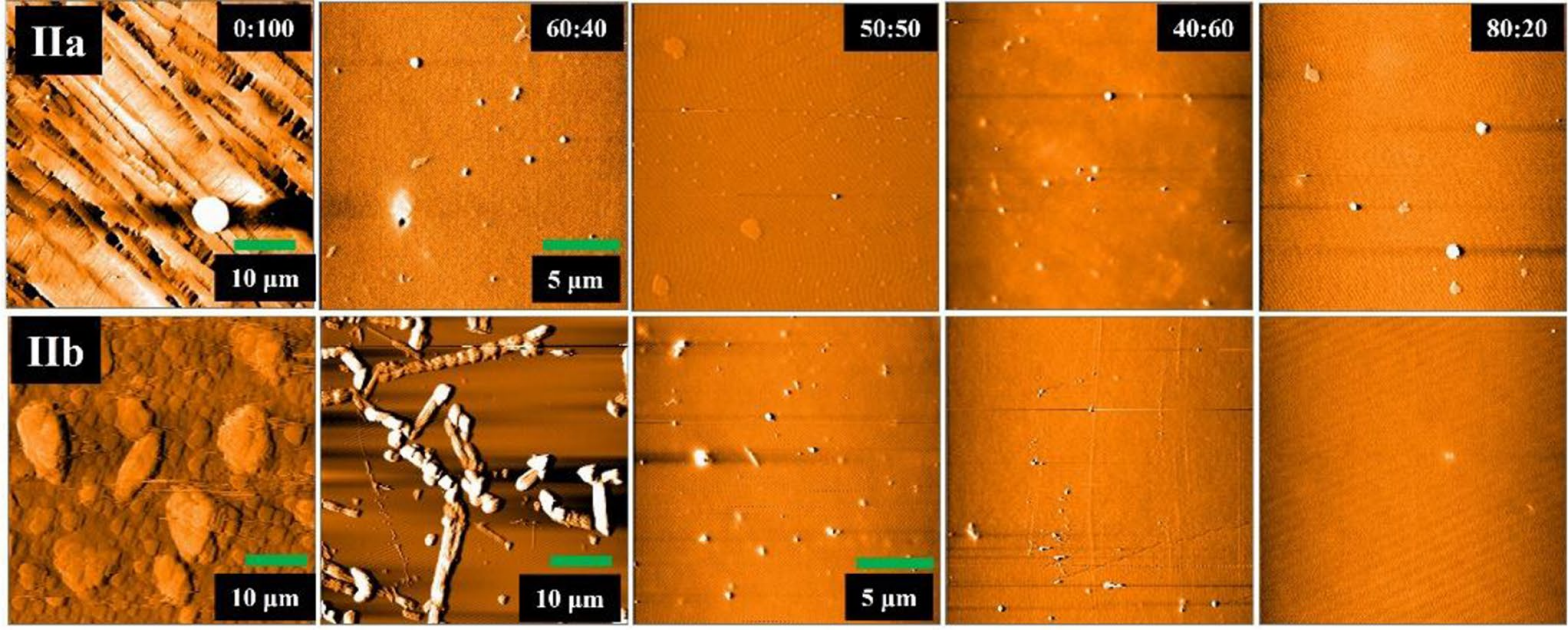
AFM images for film morphology of PVKdye **II** films, the scanned area is 20 × 20 μm, except films with higher dye concentration

The morphology of PVK:dye films was analyzed by atomic force microscopy, Figs. 8 and 9. Figure 8 shows representative images for AFM analysis of films deposited from each PVK: dye ratio. For a 0:100 ratio, aggregates of tens of micrometers are formed instead of a homogeneous film; the emission for these pure films corresponds to that of compounds in powder form. The film for the ratio 40:60 showed large aggregates segregated from the PVK film. However, in almost all cases, the emission from these films showed the redshift in emission owing to change in the intermolecular interactions of each dye. For ratios 50:50 and 60:40, smaller aggregates of several nanometers are observed on the film surface. Despite the emission of PVK-dye films agrees with emission owing to exciplex formation, the morphological analysis indicates that this is not the main emission mechanism because it is known that exciplex occurs only at the interface between both compounds. Therefore, to favors exciplex homogeneous films with domains in the range of nanometers should be formed; Benson-Smith et al. reported the importance of intimate mixing and small domain size of donor and acceptor materials to encourage exciplex formation [41]. Herein, such intimate mixing occurs only for the ratio 80:20; meanwhile, for the other ratios, segregation in the range of micrometers is observed. This suggests that for the compounds **Ia**-**Id** and **IIa**-**IIb**, the emission arise from change in the intermolecular interactions when embedded in PVK along to excitons formation at the polymer-dye interfaces.

**Fig. 10:**
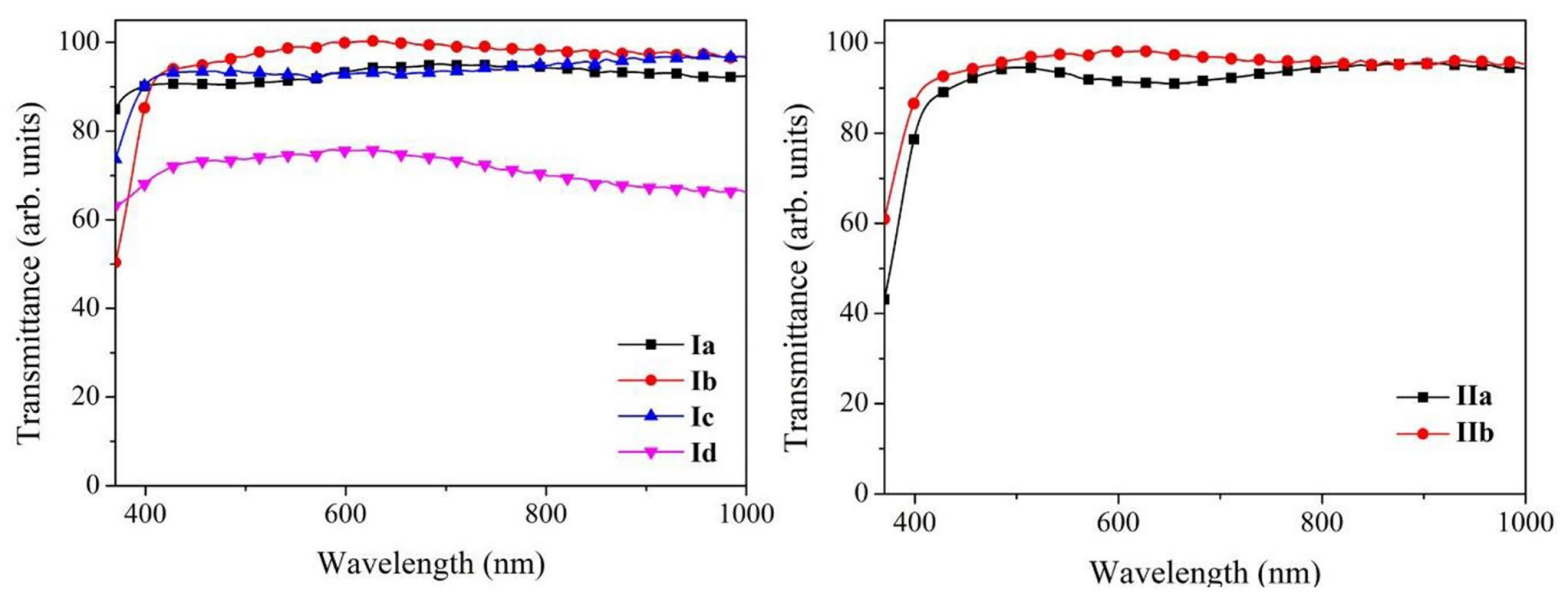
Transmittance spectra for films PVK:dye

Because the exciplex complex occurs only in the excited state, no ground state interaction between the D-A species exists, and the absorption spectrum of the blend is the sum of components itself (Fig. 10). Hence, because the absorption for the dyes and PVK is in the UV region, the films based on the PVK-dye blend are highly transparent in the visible region of the electromagnetic spectrum. The transmittance is above 90% for all the films with the 80:20 ratio, except for the blend with dye **Id**; these films showed dye segregation and looked opaque. However, the exciplex formation and the redshift in emission were observed. The high transmittance for the films could be used for transparent OLEDs or fluorescent windows.

**Fig. 11:**
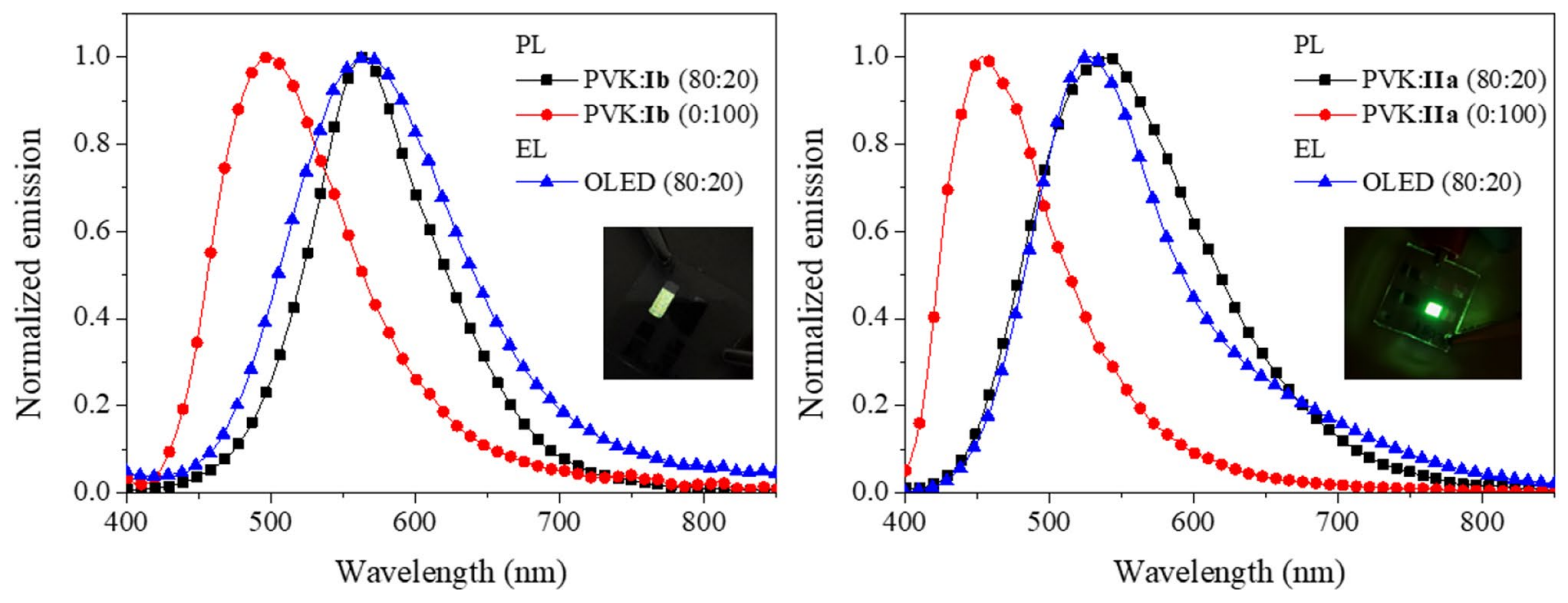
Fluorescence and electroluminescence spectra for blends PVK:**Ib** and PVK:**IIa**

Films with a ratio of 80:20, with more significant redshifts in emission and better morphology, were used as active layers in OLEDs. Figure 11 shows the comparison between PL and EL spectra for dyes **Ia** and **IIb**, which are representative of all the dyes. In both cases, the EL spectra are similar to the PL of PVK:dye blend and are red-shifted compared with pure dye. This confirms the formation of exciplex formation.

## Conclusion

Shift in fluorescence emission was achieved for films based on the blend PVK:dye owing to intermolecular interactions and exciplex formation. Several PVK: dye ratios were tried for films, and for all the ratios, the shift was observed, even in those films with high dye concentration where morphological analyses showed dye segregation from the polymer matrix. The calculated exciplex energy agrees with the film emission, which confirms the exciplex formation. Despite the non-uniform mixture in the films with higher dye concentration, the exciplex formation indicated that exciplex is energetically favored over excitons.

## Data Availability

No datasets were generated or analysed during the current study.
